# Current understanding on Retinitis Pigmentosa: a literature review

**DOI:** 10.3389/fopht.2025.1600283

**Published:** 2025-06-12

**Authors:** Naning Suleman

**Affiliations:** Department of Ophthalmology, Universitas Negeri Gorontalo, Gorontalo, Indonesia

**Keywords:** Retinitis Pigmentosa, inherited retinal dystrophy, visual impairment, clinical management, photoreceptor degeneration

## Abstract

Retinitis Pigmentosa (RP) refers to a group of inherited retinal disease, often leading to legal and sometimes complete blindness. It is estimated that over a million people are afflicted with RP all over the world and show tremendous genetic and phenotypic heterogeneity, involving over 90 genes in its causation. The present literature review intends to present a qualitative overview of the genetic mechanisms, clinical features, and diagnostic methods of RP, emphasizing genotype-phenotype correlation. Therapeutic advances in gene therapy, stem cell therapies, optogenetics, retinal prosthetics, and pharmacotherapy are reviewed, and the article notes the crucial role that next-generation sequencing has had in facilitating accurate diagnoses and tailored treatment strategies. While no curative treatments have been proposed, emerging therapeutic strategies are claimed to be effective in blunting disease advancement and maintaining or restoring visual function. Patients who are afflicted with RP often undergo a variety of both physical and mental difficulties, hence requiring several eventual, timely interventions for their social-emotional conflicts. This review aims to present insightful information about the paradigm shift in RP diagnosis and treatment, serving as a complete basis for clinicians and researchers working toward the management of retinal diseases.

## 1 Introduction

Retinitis Pigmentosa (RP) is a hereditary retinal dystrophy characterized by progressive visual loss, affecting over 1.5 million individuals worldwide (1). RP is classified as a rare disease while becoming the most common inherited retinal dystrophy IRD, with estimates for the worldwide prevalence varying from 1 in 4000 (1–3). The condition is characterized by initial symptoms of night blindness, which progresses to peripheral vision loss and ultimately leads to legal blindness by the age of 40 for many patients. The impact of RP extends beyond visual impairment, contributing to increased anxiety, depression, and social isolation among affected individuals, significantly impacting their daily lives, employment, and overall quality of life (1, 4). Furthermore, RP is linked to a higher risk of other eye complications, including cataracts and cystoid macular edema (CME), which can lead to further vision problems (5, 6).

It highlights the importance of a multidisciplinary approach in diagnosing and managing RP, emphasizing the role of genetic counseling and next-generation sequencing (NGS) in achieving an accurate diagnosis (7). Currently, a variety of management strategies for RP are available, encompassing genetic and psychological counseling as well as interventions for complications associated with RP. While these strategies are primarily supportive in nature, they contribute to alleviating the physical, psychological, and socio-emotional burdens that patients may experience (5, 8). Research on RP is significant due to its impact on quality of life and the potential for advancement in gene therapies and other treatment modalities.

This literature review aims to explore and provide an updated overview of the current RP, focusing on its genetic underpinnings, recent research advancements, genotype-phenotype correlations, therapeutic strategies, and prospects. This information can assist clinicians in offering patients the most current treatment options, helping them assess the advantages and disadvantages, and ultimately providing guidance on managing their condition.

## 2 Retinitis Pigmentosa as an inherited retinal disease

### 2.1 Genetic basis of RP

RP is primarily caused by genetic mutations affecting the function and survival of photoreceptors, particularly the rod cells, leading to significant vision impairment (3, 7). The genetic landscape of RP is remarkably complex and demonstrates extraordinary genetic heterogeneity (7, 9). RP is primarily a monogenic disease, meaning it is caused by mutations in a single gene. To date, over 90 genes have been associated with RP, and ongoing advancements in diagnostic techniques are likely to increase this number (5). RP can follow different inheritance patterns, including autosomal dominant (30-40% of cases) which is characterized by mutations in genes such as RHO, RP1, and PRPH2, also often shows variable expressivity within families and typically has a later onset and milder progression compared to other forms. Autosomal recessive (50-60% of cases) involves mutations in genes like USH2A, PDE6A, and PDE6B. Generally, this pattern presents with earlier onset and more severe progression and shows higher prevalence in populations with consanguineous marriages. X-linked (5-15% of cases) primarily affects males through mutations in RPGR and RP2 genes, demonstrates complex inheritance patterns with variable carrier effects in females, also often associated with more severe phenotypes and earlier onset (9).

Mutations in genes have been widely studied and each of it are correlates with specific disease phenotypes. More than 50 genes have been identified as causes of non-syndromic RP, with nearly 3,100 mutations documented (10). These genetic variations correlate with specific disease phenotypes, contributing to the variability in clinical presentation and disease progression (3). Specific mutations, such as those in the RHO gene associated with autosomal dominant RP and USH2A gene linked to Usher syndrome, which includes both hearing loss and RP, have been identified. Syndromic RP is characterized by additional systemic symptoms, with common syndromes including Usher syndrome, Bardet-Biedl syndrome, and Alport syndrome, which primarily affects the kidneys but can also lead to vision loss. Additionally, secondary RP occurs when other systemic diseases, such as Lyme disease, syphilis, and nephrotic syndrome, lead to retinal degeneration, manifesting symptoms similar to RP (11).

The mechanisms underlying the pathophysiology of RP involve several processes, including apoptosis of photoreceptors, dysfunction of the visual cycle, and impaired cellular signaling pathways. The initial loss of rod photoreceptors is often followed by secondary degeneration of cone photoreceptors, leading to significant visual impairment. The disease mechanisms can vary depending on the specific genetic mutations involved, with some mutations affecting protein folding, trafficking, or function, ultimately leading to photoreceptor cell death (12). The identification of these mutations has been greatly aided by advances in genetic testing technologies, such as NGS, which allows for comprehensive screening of retinal dystrophy genes. Understanding the specific genetic mutations involved offers crucial insights into the pathophysiology of RP and potential therapeutic strategies (3).

### 2.2 Clinical presentation

Clinically, RP presents a range of symptoms, beginning with night blindness (nyctalopia), which is the initial indication of the condition. This difficulty in adjusting to darkness occurs because the rod cells are responsible for low-light vision. This results in “tunnel vision,” where peripheral vision is lost, and individuals can no longer see objects to the side without turning their heads. As the disease progresses, patients experience a gradual loss of central vision, affecting their ability to perceive details and colors, especially green and blue. Photophobia, or excessive sensitivity to light, also develops as cone cells are impacted, complicating vision under bright lighting (1). However, the severity and onset of symptoms can vary significantly among individuals, even within the same family, highlighting the unpredictable nature of the disease (5).

**Figure 1:**
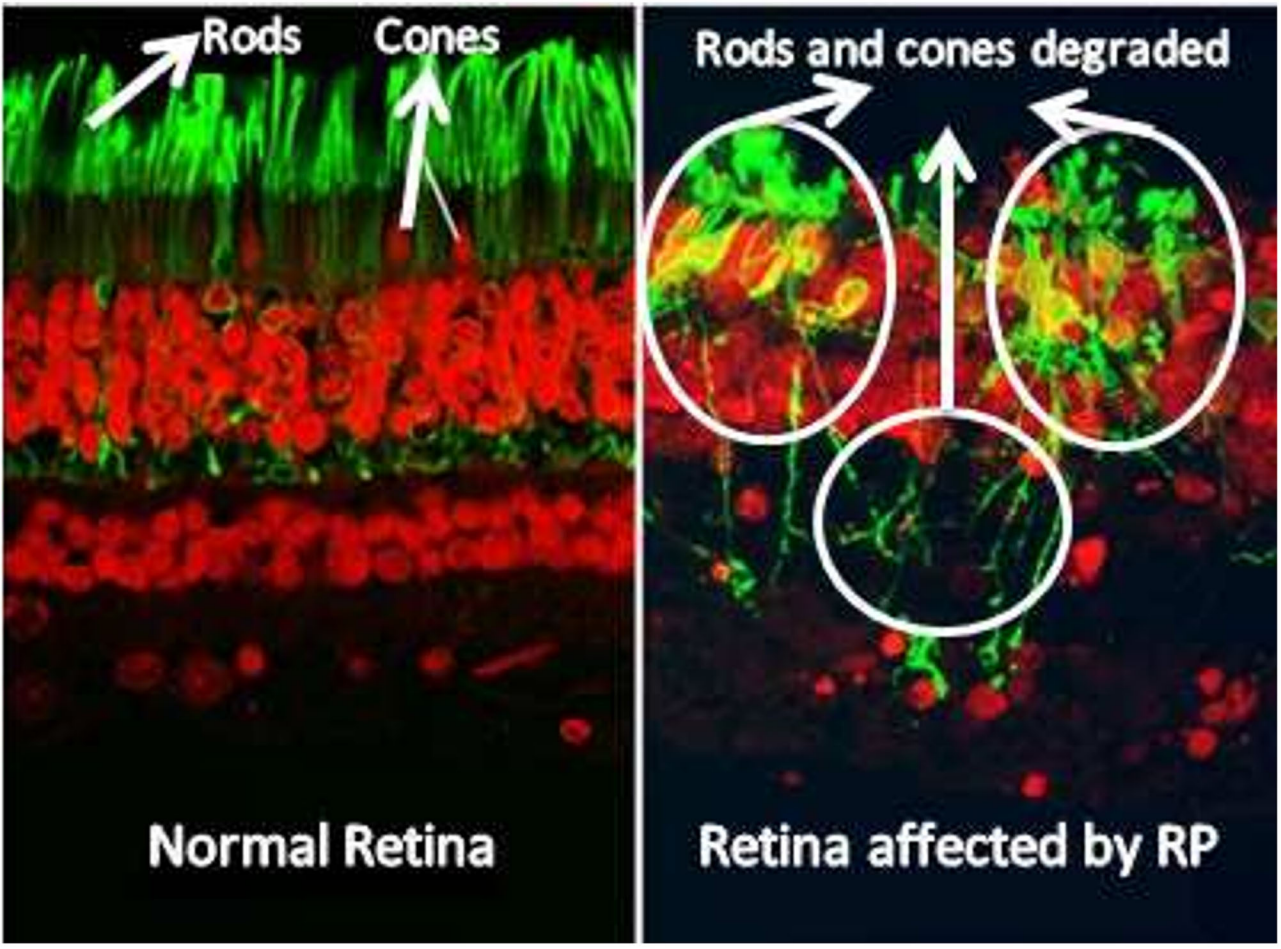
Retina image of normal retina and patient with retinitis pigmentosa. The retina affected by retinitis pigmentosa shows reduced density of rod and cone cells (13).

**Figure 2:**
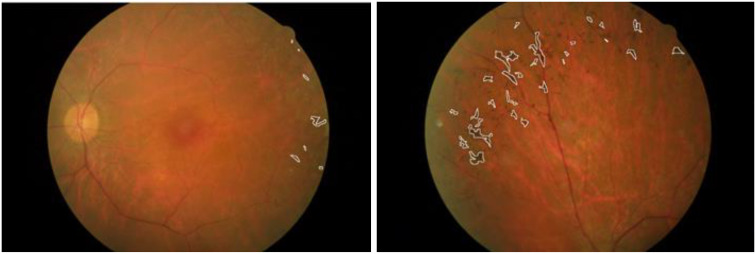
Fundus images of a patient with retinitis pigmentosa taken from different angles (13).

**Figure 3:**
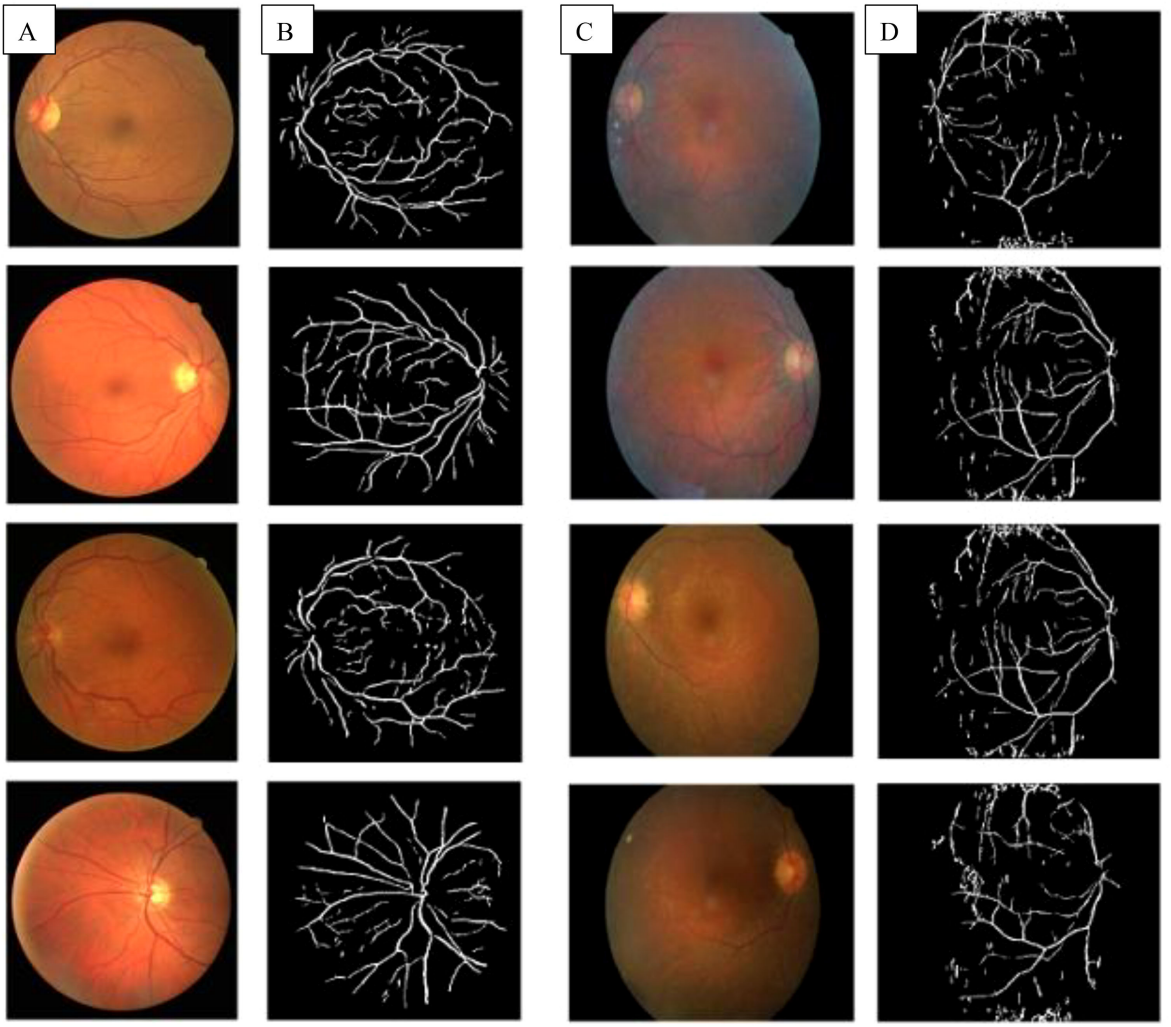
**(A)** Fundus images of healthy controls; **(B)** Extracted blood vessels from healthy controls; **(C)** Fundus images of pigmentosa patients showed waxy pallor of optic nerve head; **(D)** Extracted blood vessels from retinitis pigmentosa patient showed attenuation of the retinal vessel (13).

In a healthy retina, the architecture of rod and cone photoreceptors is orderly; however, in patients with retinitis pigmentosa (RP), one observes notable irregularities and potential loss of the rod and cone cell layer. Significant differences in retinal structure between normal individuals and those with RP are illustrated in **Figure 1**. Fundus examination reveals characteristic findings, including bone spicule pigmentation, attenuation of retinal vessels, and waxy pallor of the optic nerve head, as depicted in **Figure 2**. These alterations in fundoscopic images are accompanied by vascular changes, specifically a reduction in blood vessel density in cases of RP, as demonstrated in **Figure 3**. In the later stages, cataracts may develop, further impairing vision. Visual acuity may decline significantly, and patients often report difficulties adapting to changes in lighting or weather conditions (1). The variability in clinical presentation is influenced by the specific genetic mutations involved, with some patients experiencing more rapid progression than others (3).

RP is associated with several ocular complications that can significantly impact the visual function and quality of life of affected individuals. One of the most common complications is central macular edema (CME), characterized by the accumulation of fluid in the macula, which can lead to significant central vision loss. Another prevalent complication is cataracts, particularly posterior subcapsular cataracts, which can develop at an earlier age in RP patients compared to the general. Glaucoma is also a concern, as RP patients are at a higher risk of developing this condition, which damages the optic nerve and can lead to peripheral vision loss. Regular monitoring of intraocular pressure and visual fields is essential, with treatment options including topical medications, laser therapy, or surgical interventions. Other complications include night blindness, and retinal detachment, particularly in patients with high myopia (11, 14).

## 3 Basic, clinical, and translational studies

### 3.1 Basic research

RP is characterized by the progressive loss of retinal photoreceptors, primarily affecting rod cells followed by cone cells. The mechanisms underlying photoreceptor degeneration in RP are complex and multifactorial (15). Research into the mechanisms of photoreceptor degeneration in RP has identified several pathways involved, including oxidative stress and apoptosis. Apoptosis, a form of programmed cell death, involves intrinsic and extrinsic pathways that activate caspase proteins, leading to cell fragmentation. Recent findings suggest that regulated necrosis, including necroptosis, ferroptosis, and parthanatos, may also contribute to RP progression by causing inflammation and tissue damage (15). In addition, mitochondrial dysfunction and endoplasmic reticulum (ER) stress are commonly observed in photoreceptor cells during RP, triggering apoptosis and further cellular degeneration (16). Metabolic dysfunctions, including disruptions in glucose metabolism, further exacerbate retinal degeneration by reducing the energy supply required for photoreceptor survival (17).

Oxidative stress is a significant factor in RP pathogenesis. Photoreceptors are highly metabolically active and are therefore prone to oxidative damage due to reactive oxygen species (ROS) generated during normal cell activity and exacerbated by degeneration (15). Studies have shown that oxidative stress leads to cell death by disrupting the balance between ROS production and antioxidant defense mechanisms, contributing to retinal damage and disease progression (18). The accumulation of ROS can cause damage to cellular components, including lipids, proteins, and nucleic acids, ultimately leading to photoreceptor cell death (19). Additionally, calcium dysregulation and endoplasmic reticulum stress play roles in photoreceptor death, as the accumulation of misfolded proteins activates the unfolded protein response, which can lead to apoptosis if homeostasis is not restored (15). Understanding the interplay between these pathways, as well as the role of microglia in inflammation, is crucial for developing therapeutic strategies for RP and other retinal diseases (15).

Microglia, the resident immune cells of the central nervous system, play a crucial role in the retinal response to injury and disease. In the context of RP, microglial activation is a significant aspect of the disease progression. Under normal conditions, microglia maintain homeostasis and support neuronal health. However, in response to oxidative stress and retinal degeneration, microglia become activated and can adopt a pro-inflammatory phenotype. Activated microglia release various inflammatory mediators, including cytokines and chemokines, which can further exacerbate oxidative stress and promote photoreceptor cell death. This neuroinflammatory response creates a vicious cycle, where oxidative damage leads to microglial activation, which in turn contributes to further oxidative injury and retinal degeneration (19).

Retinal metabolic disorders, resulting from impaired energy supply to photoreceptors, contribute to photoreceptor death and exacerbate degeneration (19). Animal models have provided significant insights into these processes and have been instrumental in testing potential treatments (18). These various animal models, including rodents, dogs, cats, pigs, and non-human primates, have helped identify key molecular pathways and aided in the development of targeted therapies for RP.

Studies on animals, particularly mammals, have further shed light on the molecular pathophysiology of RP. **Table 1** summarizes the animal models used to study RP and its findings.

**Table 1:** Animals models in the study of RP (20, 21).

Animal model	Strain	Gene	Description
Rat	P347L	RHO	Substitution of proline with leucine at position 347 of RHO resulted in rapid degeneration of outer nuclear layer of photoreceptor cells shortly after birth allowing for quicker assessments of drug efficacy compared to other animal models that exhibit slower disease progression
Mice	Rho-P23H Rho-T17M		These strains exhibited mutations associated with autosomal dominant RP.
	Rpe65−/−	RPE65	Mice with homozygous non-functional RPE65 are usually used to study Leber congenital amaurosis, but also provide insights into RP due to the involvement of RPE65 in the visual cycle
Dog			Same as mice. The RPE65 dog model was instrumental in the first FDA-approved gene therapy for retinal disease. Various breeds also exhibit inherited retinal diseases, including progressive retinal atrophy which has similarities with RP.
Cats	–	CEP290	These models exhibit retinal degeneration patterns that closely resemble human diseases.
		CRX	
Pigs	Pro23His	RHO	Transgenic pigs have been developed to study RP, particularly those with mutations in the RHO gene. The porcine eye size and structure is suitable for surgical interventions and drug delivery studies
Primates	Various	Various	These models are valuable for studying retinal diseases due to their closer anatomical and functional similarities to human retinas.

### 3.2 Clinical examination

Longitudinal studies of RP progression have proven vital in understanding the disease course. Such studies reveal a consistent decline in visual acuity and visual field as the disease progresses, with notable variability across genotypes (10). These studies consistently show a decline in visual acuity and visual field over time. A large-scale study of sector RP, a subtype of RP with limited retinal degeneration, indicated that patients maintain good central vision despite disease progression, though retinal deterioration continues over time, as evidenced by serial fundus autofluorescence (FAF) imaging (16). This highlights the importance of regular monitoring for early detection of disease changes.

The role of retinal imaging techniques such as optical coherence tomography (OCT) and FAF play a pivotal role in diagnosing RP and tracking its progression. These technologies allow for the visualization of photoreceptor loss, retinal thinning, and other structural changes. Electroretinography (ERG) is also used to assess rod and cone photoreceptor function, helping to stage the disease and predict prognosis (16). Genetic studies have identified strong correlations between specific mutations and clinical manifestations of RP, such as mutations in RHO, RPGR, and PRPS1 genes, which affect inheritance patterns and disease severity (17).

#### 3.2.1 Optical coherence tomography

**Figure 4:**
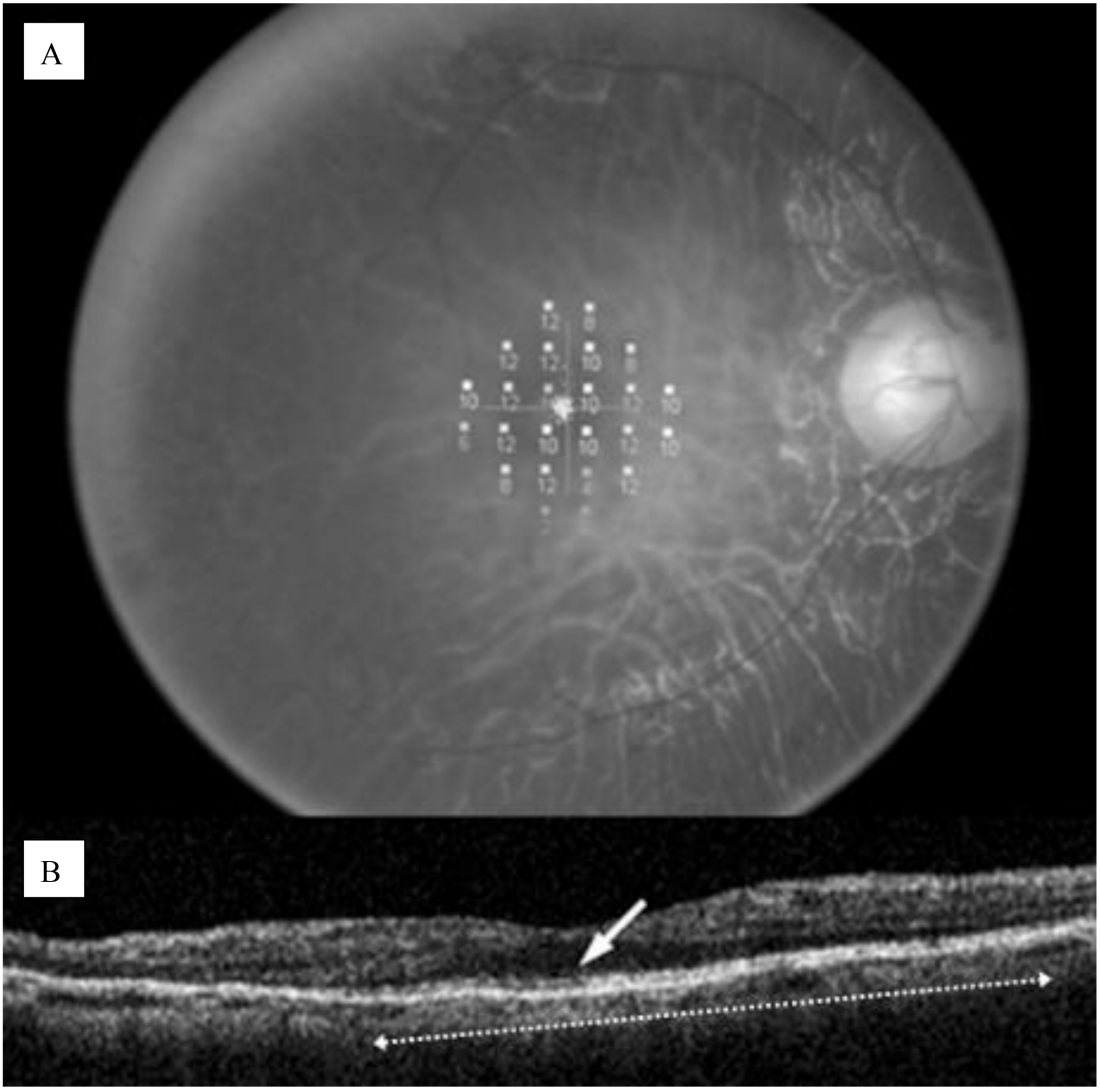
**(A)** Microperimetric map image of a 60-year-old man with retinitis pigmentosa; **(B)** OCT image of a 5-mm horizontal scan, the IS/OS line can be observed as a distinct highly reflective line (white row), the length was computed to be 3.6 mm (white dotted arrow. Disruption of the IS/OS line indicates photoreseptor degeneration (22).

One of the key imaging techniques is OCT, a non-invasive method that provides high-resolution cross-sectional images of the retina. OCT allows for the visualization of retinal layers and the assessment of photoreceptor integrity. A critical marker of photoreceptor health is the photoreceptor inner/outer segment (IS/OS) junction, whose presence and continuity correlate with visual function (22). An intact IS/OS line is associated with better visual acuity, while its disruption indicates photoreceptor degeneration. The inner, outer, and total retinal thickness along with examination of the ellipsoid zone band can also be evaluated with OCT, as shown in **Figure 4**. Additionally, it can measure foveal thickness, which is often reduced in RP patients due to photoreceptor loss, providing insights into disease progression and the effectiveness of potential therapies (22, 23).

However, OCT has several limitations in monitoring RP. The axial resolution of spectral-domain OCT, which is typically 1–10 μm may be inadequate in detecting small changes. OCT may also be unsuitable for patients with advanced disease as good fixation may be difficult due to poor vision, hindering an appropriate assessment. Technological advancements have made OCT less time-consuming without sacrificing consistency with the development of software to automate the segmentation of retinal architecture, though this may be challenging in RP patients with significant changes to retinal architecture. Studies have also utilized retinal thickness as measured by OCT to evaluate therapeutic response in the treatment of RP (23).

#### 3.2.2 Fundus autofluorescence

**Figure 5:**
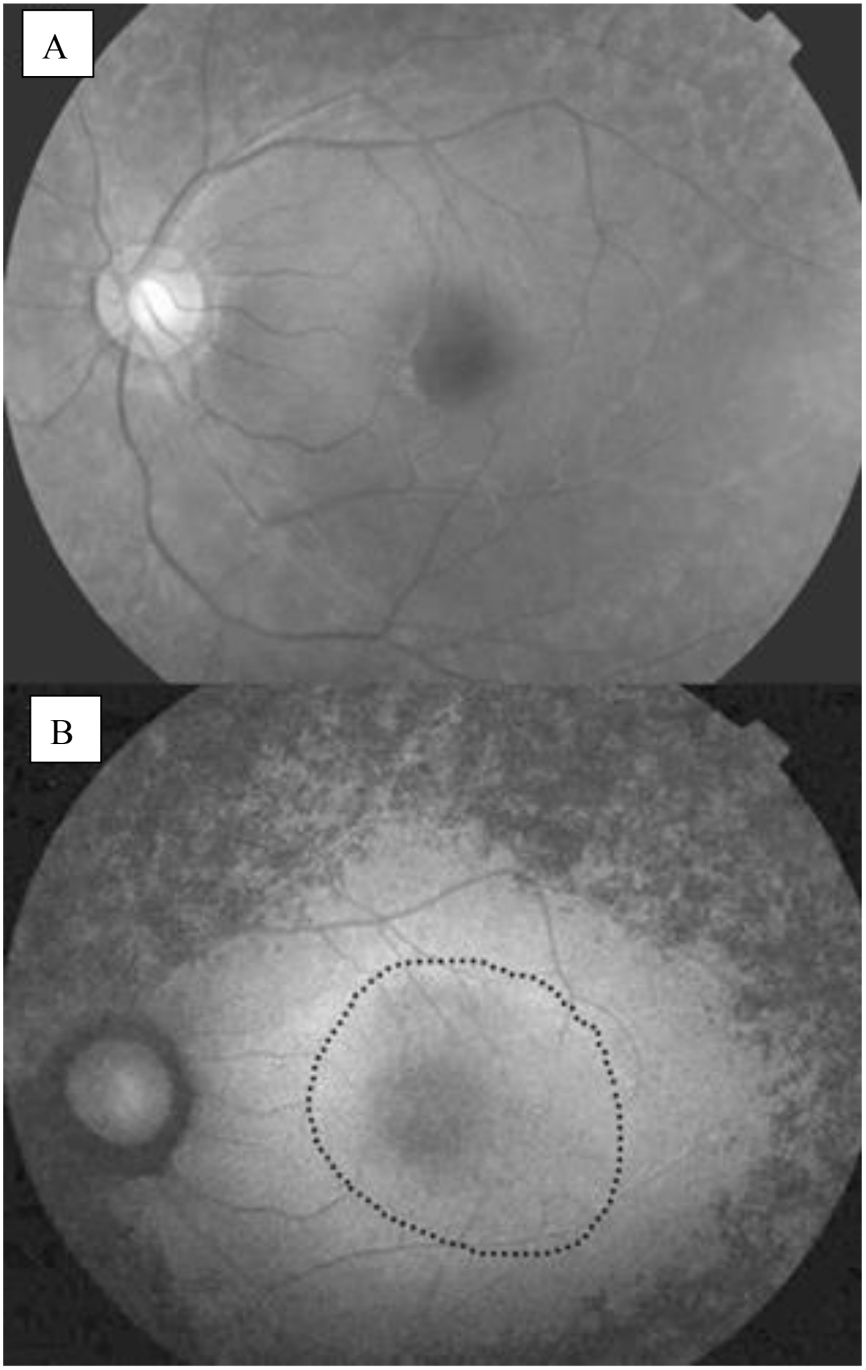
**(A)** Normal fundus photograph; **(B)** Fundus autofluorescence image of an eye with RP, an abnormal parafoveal ring of high density fundus autoflourescence (black dotted circle) (22).

FAF is a non-invasive imaging technique that provides insights into the health of the retinal pigment epithelium (RPE) and photoreceptors by visualizing the autofluorescent properties of certain retinal components. In the context of RP, FAF can reveal characteristic patterns associated with the disease, helping to assess the extent of retinal degeneration and monitor disease progression (24). Many RP patients exhibit an abnormal parafoveal autofluorescence ring, representing the border between functional and dysfunctional retina, as shown in **Figure 5**. The diameter of the AF ring correlates with retinal sensitivity and visual function, while different FAF patterns can indicate varying degrees of retinal degeneration. For instance, a high-density autofluorescence ring may suggest active photoreceptor degeneration, whereas decreased or absent FAF may indicate RPE atrophy (22). It was also shown that the presence of the AF ring in RP is correlated with loss of the ellipsoid zone, but not the external limiting membrane (25).

#### 3.2.3 Electroretinography

The ERG is a crucial diagnostic tool used to evaluate retinal function in patients with RP. It measures the electrical responses of photoreceptors in the retina when exposed to light, providing valuable insights into retinal dystrophies. The interpretation of ERG results focuses on the amplitudes and implicit times of the a-wave and b-wave, where decreases in amplitude indicate photoreceptor damage, and delays in implicit time suggest overall impairment in photoreceptor function. Additionally, ERG can be utilized to predict visual prognosis in RP patients, with a decline in the 30-Hz cone ERG amplitude serving as an indicator of remaining visual function. In order to obtain a valid and accurate measurement, it is imperative for the electrodes to be properly placed. They should also be cleaned after use as precaution to infection (26). The International Society for Clinical Electrophysiology of Vision regularly releases a standard ERG protocol, with the latest being published in 2022 (27).

**Figure 6:**
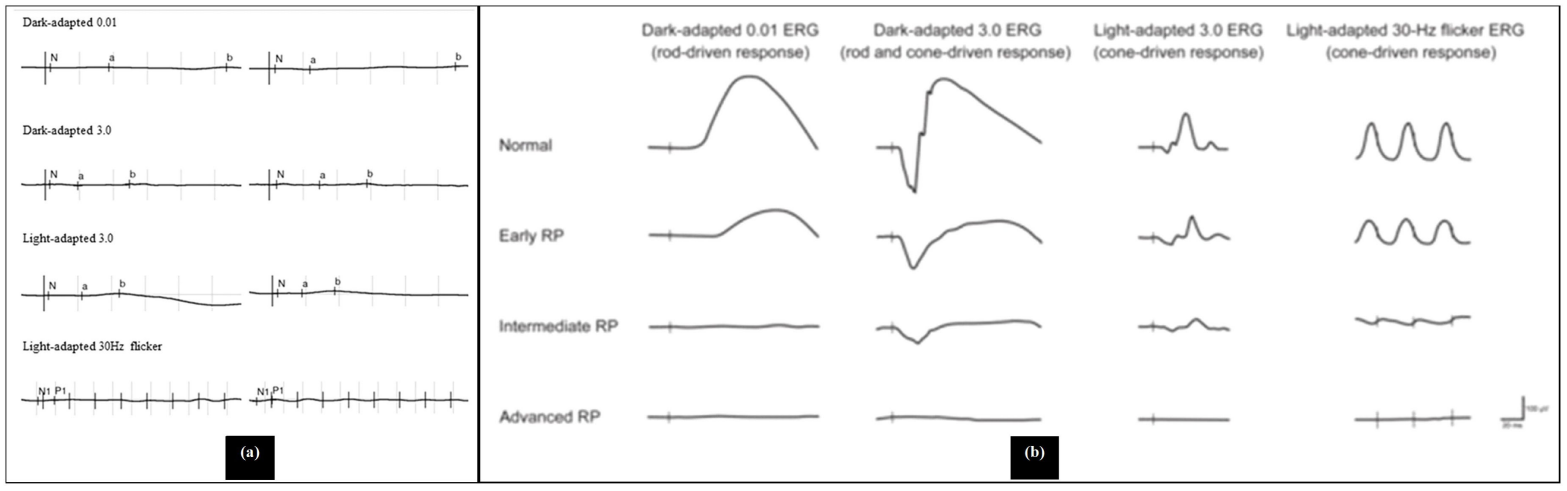
Abnormal electroretinogram results, **(a)** Electroretinography results/electroretinograms of patients with retinitis pigmentosa (a young patient with cystoid macular edema), **(b)** electroretinogram recordings in early, intermediate and advanced stages of retinitis pigmentosa (28, 29).

**Figure 7:**
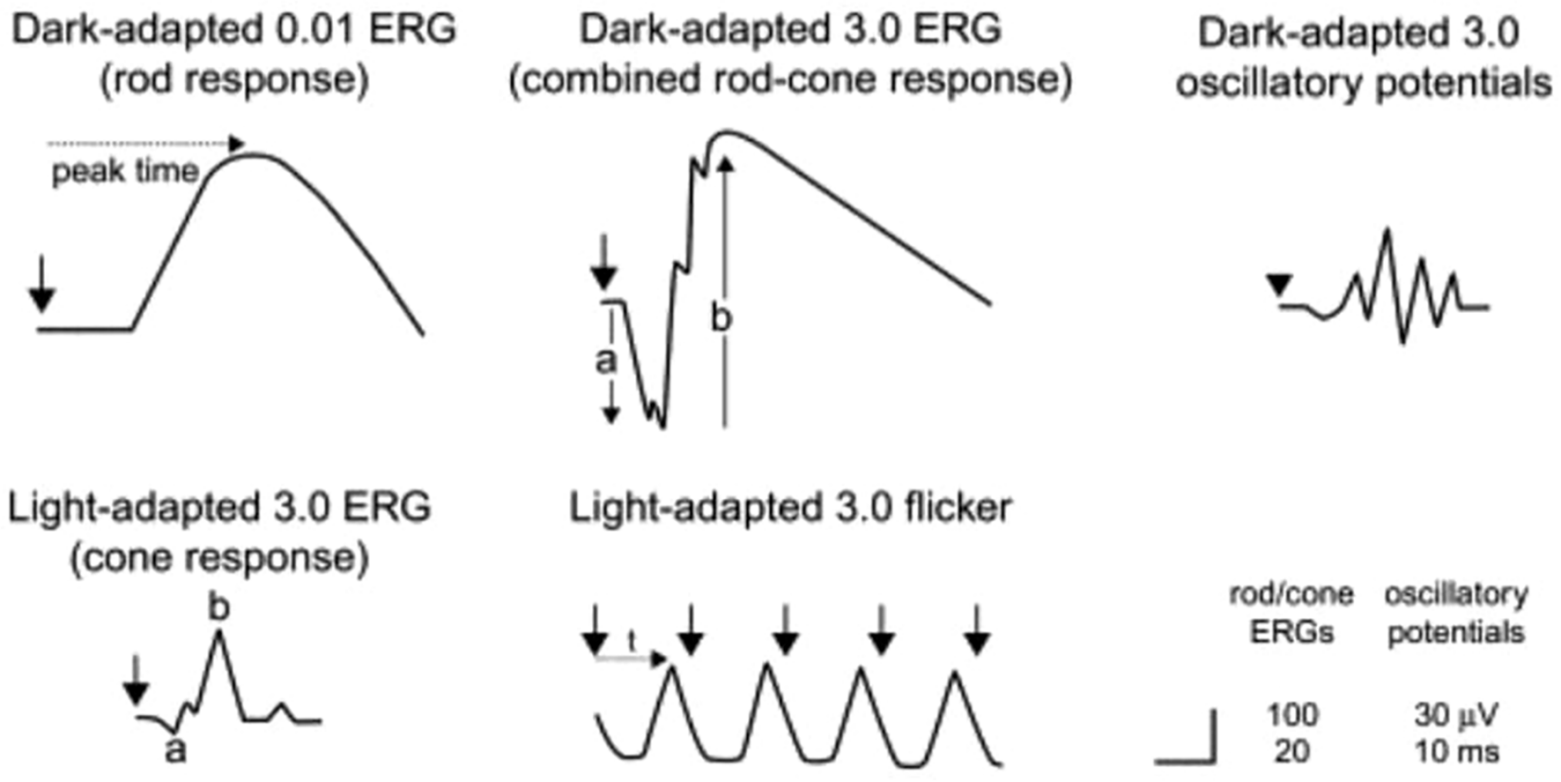
Electroretinography results/electroretinograms of healthy individual (29).

In the early stages of RP, rod photoreceptors are primarily affected, leading to reduced b-wave amplitudes and delayed implicit times in the dark-adapted 0.01 ERG, which assesses rod function. As the disease progresses, both rod and cone functions deteriorate. The dark-adapted 3.0 and 10.0 ERGs show decreased a-wave amplitudes, indicating impaired rod photoreceptor function, while the b-wave amplitudes are also reduced due to the abnormal connections with ON-bipolar cells. Light-adapted ERGs, such as the 30-Hz and 3.0 ERGs, typically exhibit delayed and/or reduced responses from cone photoreceptors, although cone function is generally less affected than rod function (26). **Figure 6** illustrates the explanation of the ERG of RP patients. As a comparison, **Figure 7** shows the pattern of normal ERG recording.

### 3.3 Translational research

Recent advancements in gene therapy have shown great promise for treating RP. One of the leading approaches is the use of adeno-associated virus (AAV) vectors to deliver functional copies of mutated genes. The Luxturna trial, which focuses on RPE65 gene therapy, has demonstrated success in restoring vision and halting disease progression in certain RP subtypes (30). These ‘first-in-human’ trials demonstrate the potential to restore vision or halt disease progression in specific subtypes of RP, offering hope for patients. In addition to gene therapy, researchers are exploring combination therapies that combine gene therapy with pharmacological treatments to enhance therapeutic efficacy. Neuroprotective strategies, such as the use of rod-derived cone viability factor, have also been investigated to prevent secondary degeneration of cones, offering additional therapeutic potential (17). Gene therapy clinical trials, including those targeting RHO-related RP, have shown encouraging results, with improvements in both visual acuity and retinal function following treatment (18). These developments, alongside ongoing research into neuroprotective approaches, offer new hope for patients suffering from RP and other retinal diseases. Current efforts are also exploring combination therapies that utilize gene therapy alongside pharmacological approaches to enhance efficacy and manage disease.

## 4 Genotype and phenotype studies

### 4.1 Genotype-phenotype correlations

Understanding the correlation between genotype and phenotype in RP is essential for precise diagnosis and personalized treatment. Different mutations can result in varying degrees of severity, progression rates, and associated symptoms such as hearing loss in syndromic RP (e.g., Usher syndrome). Large-scale genetic screening and whole-genome sequencing have facilitated the identification of novel RP-related mutations. Recent work on PRPF31 mutations has revealed that truncating variants (e.g., frameshift and nonsense mutations) are often associated with an earlier onset and more severe retinal degeneration, emphasizing the role of haploinsufficiency and dominant-negative effects in disease progression (31). This detailed genetic characterization is critical for predicting patient prognosis and designing targeted therapeutic strategies (31).

Parallel investigations into PRPH2-related diseases have illustrated similar correlations, where the functional impact of missense variants—as determined by multiple in silico prediction tools—is directly linked to the specific retinal phenotype observed. For instance, patients harboring certain damaging missense variants are more likely to develop classic RP, while others may present with macular dystrophies, thereby reflecting the diverse outcomes based on genotype (32). These findings underscore the importance of a comprehensive variant assessment for accurately predicting the clinical course of RP (32).

Studies focusing on RP1-associated retinal dystrophies in Japanese cohorts further enrich the genotype-phenotype narrative. Analysis of RP1 mutations has demonstrated that both the type (truncating vs. missense) and the location of the variant can dictate the inheritance pattern and subsequent disease progression, with autosomal recessive cases often exhibiting a more severe clinical course than autosomal dominant ones (33). Such correlations emphasize the heterogeneity of RP and highlight the necessity for integrating genetic screening into routine clinical practice to better predict outcomes and tailor management plans (33).

### 4.2 Various phenotypes

RP is characterized by a broad clinical spectrum, ranging from the classical form—with night blindness and peripheral visual field loss—to atypical presentations that may involve central macular degeneration. In patients with PRPF31 mutations, this variability is often attributed to incomplete penetrance and variable expressivity, where even individuals sharing the same pathogenic variant can display remarkably different disease severities (31). This phenotypic diversity complicates diagnosis and calls for personalized clinical approaches to manage RP effectively (31).

Research on PRPH2-associated retinal dystrophies further highlights the diversity in clinical phenotypes observed among different populations. For example, in a large Chinese cohort, RP was the predominant presentation, whereas literature from predominantly Caucasian groups has reported a higher incidence of macular degeneration in patients with PRPH2 mutations (32). These ethnic-specific differences suggest that genetic background and potential modifier factors play a crucial role in the phenotypic manifestation of retinal dystrophies (32).

In addition, studies on RP1-associated retinal dystrophies in Japan reveal that the clinical features can vary according to the underlying inheritance pattern. Patients with autosomal dominant RP1 mutations often experience a more gradual disease progression, while those with autosomal recessive variants tend to have an earlier onset and faster deterioration of visual function (33). These observations provide important insights into the spectrum of RP phenotypes and stress the need for genetic information to be considered when prognosticating disease outcomes and formulating management strategies (33).

### 4.3 Emerging technologies

Recent advances in NGS have revolutionized the field of retinal genetics by enabling rapid and comprehensive identification of pathogenic variants linked to RP. High-throughput sequencing methods, including whole-exome and whole-genome sequencing, have been instrumental in mapping the complex genetic landscape of RP and have greatly improved diagnostic precision (33). This technological progress not only accelerates variant discovery but also supports the development of genotype-specific therapeutic interventions (33).

Beyond sequencing, stem cell-based models—particularly patient-derived induced pluripotent stem cells and retinal organoids—are emerging as powerful tools to study the underlying molecular mechanisms of retinal degeneration. These *in vitro* models have allowed researchers to replicate key aspects of RP pathology, including aberrant splicing defects in retinal cells, and provide a platform for high-throughput drug screening and functional studies (31) By recapitulating patient-specific phenotypes in a controlled environment, these models offer promising avenues for personalized medicine and the validation of novel therapeutic targets (31).

Emerging therapeutic strategies such as gene augmentation and antisense oligonucleotide (AON) therapies are also gaining momentum as potential treatments for RP. Recent studies have explored the feasibility of correcting pathogenic mutations using genome editing tools like CRISPR/Cas9, which holds promise for permanent gene correction and long-term rescue of retinal function (32). Collectively, these innovative diagnostic and therapeutic technologies are set to transform the clinical management of RP, paving the way for precision medicine in retinal disorders (32).

## 5 Therapy

Current therapeutic strategies primarily focus on gene therapy, which aims to correct genetic defects associated with RP. Various approaches have been explored, including gene enhancement or replacement, gene suppression, and gene editing (9). It focuses on halting disease progression, restoring vision, or compensating for visual impairment for RP patients.

### 5.1 Gene therapy

Gene therapy has shown promise, with clinical trials demonstrating the efficacy of AAV-mediated gene delivery in restoring retinal function. The approval of voretigene neparvovec for patients with biallelic RPE65 mutations marks a significant milestone in RP treatment (7). This therapy has shown significant improvements in visual function in clinical trials and is currently the only approved gene therapy for inherited retinal diseases, specifically targeting the genetic defect rather than just alleviating symptoms (9). Many trials of gene therapy both in animal models and humans with good safety profiles and good results in clinical trials. However, there are still some disadvantages, including heterogeneity of the disease, necessity to genotype the patient, non-advanced-stage disease, and limited duration of positive effect (34). Gene therapy is a broad category that encompasses various strategies aimed at correcting or compensating for genetic defects. Within gene therapy, there are several specific approaches, including:

#### 5.1.1 Gene enhancement/replacement

This strategy is employed primarily for recessive forms of RP, where the disease results from a loss of function of a specific protein. The approach involves supplementing the affected cells with an additional copy of the wild-type gene to restore normal protein levels (9). This involves introducing a healthy copy of the gene into the retinal cells, restoring the production of the necessary protein for photoreceptor function (35). This can be achieved using viral vectors to introduce the gene into the cells. In cases where dominant-negative mutations are present, increasing the ratio of wild-type to mutant proteins may help alleviate symptoms, although it may not provide a complete cure (9). Major challenges include the variability in treatment efficacy due to the heterogeneous nature of RP, the need for precise targeting of the affected retinal cells, and the potential for immune responses against the viral vectors (14).

#### 5.1.2 Gene suppression

Gene suppression targets and inhibits the production of aberrant proteins caused by dominant-negative or gain-of-function mutations. This can be done at the DNA level through transcriptional repression or at the RNA level using techniques like RNA interference (si/shRNA) to downregulate translation. The aim is to prevent the production of harmful proteins that contribute to photoreceptor cell death, thereby preserving retinal function (9).

#### 5.1.3 Gene editing

Gene editing represents a cutting-edge approach that allows for the precise correction of mutations at the DNA level. Techniques such as CRISPR/Cas9 enable the introduction of double-strand breaks in the DNA, which can then be repaired using donor DNA templates to correct the genetic defect. This method offers the potential for permanent correction of the underlying genetic cause of RP, making it particularly applicable for autosomal dominant disorders where disrupting the mutant allele can restore normal function (35). Gene editing is particularly advantageous for addressing loss-of-function mutations that lead to complete or near-complete absence of protein function. For example, mutations in genes like RPGR and PDE6B, which are commonly associated with RP, can be targeted for correction (14). Gene editing holds great promise for future therapeutic applications in RP, although challenges such as off-target effects and delivery methods need to be addressed (9, 35).

The gene therapies described above are adjusted according to the mutation type (**Table 2**).

**Table 2:** Mutation types and their appropriate gene therapies (9).

Mutation type	Description	Gene therapy strategy	Example gene
Loss-of-function	Results in a nonfunctional gene product with the remaining copy of the gene unable to sustain normal phenotypic expression.	Gene enhancement or gene replacement	RPE65, PRPF31
		Gene editing	
Dominant-negative	Generates a mutant protein that impairs the function of the wild-type protein.	Gene enhancement or gene replacement	RP1
		Gene editing	
		Gene suppression or gene inactivation	
Gain-of-function	Introduces a deleterious function to the protein within the cell	Gene suppression or gene inactivation	RHO
		Gene editing	

### 5.2 Retinal prosthetics

This therapy employs devices that stimulate the retina to generate visual signals, bypassing damaged photoreceptors, and shows promise for restoring vision RP patients. Epiretinal devices, like Argus II and EpiRet3, can induce phosphenes but are linked to serious adverse effects, such as retinal tears and inflammation. In contrast, subretinal devices, including the Retina Implant AG’s device and the Optobionics Artificial Silicon Retina (ASR), offer higher visual resolution and fewer complications, enabling patients to recognize letters and objects. The ASR has also demonstrated unexpected neurotrophic effects, significantly improving visual acuity and color perception, suggesting potential restoration of lost visual function beyond simple retinal stimulation (36). Continued development of these technologies promises to enhance the quality of life for RP patients, though further research is necessary to optimize their effectiveness and address challenges in retinal degeneration.

### 5.3 Pharmacological interventions

The use of drugs to manage symptoms or slow disease progression, rather than correcting genetic defects, also including neuroprotective agents and vitamin A supplementation, aims to slow photoreceptor degeneration (7, 37). Vitamin A palmitate plays a crucial role in the visual cycle as its principal element, facilitating the conversion of light into visual signals. This therapy is advantageous due to its easy administration and low cost, making it accessible for many patients. Additionally, high doses of vitamin A palmitate can be teratogenic, posing risks during pregnancy. As such, while vitamin A palmitate can be a beneficial component in managing certain visual disorders, its limitations and potential side effects must be carefully considered (34). Omega-3 fatty acids have also been studied, but evidence supporting their benefit in RP is limited (38).

### 5.4 Stem cell therapy

The use of stem cells to regenerate or replace damaged retinal cells with healthy stem cells aims to restore function (7, 34, 37). It has advantages in good safety profile particularly for improvement in visual quality. On the other hand, the main challenges for stem cell therapy include ensuring proper differentiation of stem cells into functional photoreceptors, integrating these cells into the existing retinal architecture, and addressing safety concerns such as tumorigenesis. Additionally, the timing of stem cell transplantation is critical, as it is most effective before significant retinal degeneration occurs (14). This type of therapy becomes explorer as a potential future treatment, offering hope for vision restoration, and requires further research to establish safety and efficacy (7, 34, 37).

### 5.5 Optogenetics

Optogenetics is an innovative therapeutic approach that aims to restore vision in patients with RP by using light-sensitive proteins to stimulate remaining retinal cells. This technique involves the introduction of opsins, which are light-sensitive proteins, into the surviving retinal neurons, allowing them to respond to light and generate visual signals. The primary goal of optogenetics is to bypass the damaged photoreceptors that are characteristic of RP and directly activate the downstream retinal circuitry (35). The effectiveness of these therapies depends on the type of opsin, delivery method, and targeted retinal cells. However, challenges persist in optimizing opsin delivery, ensuring long-term treatment stability, and achieving vision resolution comparable to natural sight (35). Ongoing research aims to enhance the efficacy of optogenetic therapies and develop improved delivery systems, making optogenetics a promising approach for treating retinal degenerative diseases and improving visual outcomes for RP patients.

## 6 Conclusion

RP is still among the most common causes of inherited blindness with profound and far-reaching implications affecting not only the patient’s vision but also their quality of life and psychological well-being. It requires a multidivisional approach in both its diagnosis and management. The genetics of RP are very complex and require advanced diagnostic methodologies like genetic screening and NGS for the effective tailoring of early treatment options in patients. Genetic counseling also plays a crucial role in the management of RP, as it helps affected individuals and their families understand the inheritance patterns, potential risks for future generations, and available testing and therapeutic options. To date, no definitive cure for RP exists, but much progress has been made in gene therapy, stem cell transplants, neuroprotective agents, and retinal prosthetics which offer avenues by which disease progression may be slowed and visual function might be restored. Optogenetics and bioengineered retinal implants indicate the undertaking of engaging and novel practices that may transform treatment paradigms of the future. The present investigation must address emerging therapies and their optimization, the efficacy of the treatments, long-term safety, and accessibility for the patients. Moving forward, a combination of approaches tapping upon molecular genetics, clinical ophthalmology, and high-end biomedical innovations will be most relevant in providing hope for interventions that may slow disease progression or restore vision in individuals with RP.
